# Machine learning prediction of sleep stages in dairy cows from heart rate and muscle activity measures

**DOI:** 10.1038/s41598-021-90416-y

**Published:** 2021-05-25

**Authors:** Laura B. Hunter, Abdul Baten, Marie J. Haskell, Fritha M. Langford, Cheryl O’Connor, James R. Webster, Kevin Stafford

**Affiliations:** 1 Animal Behaviour and Welfare, AgResearch Ltd., Ruakura Research Centre, Hamilton, Waikato New Zealand; 2 Bioinformatics and Statistics, AgResearch Ltd., Grasslands Research Centre, Palmerston North, Manawatu New Zealand; 3 Department of Animal Science, School of Agriculture and Environment, Massey University, Palmerston North, Manawatu New Zealand; 4 Animal Behaviour and Welfare, Scotland’s Rural College (SRUC), Edinburgh, Scotland, UK; 5 Institute of Precision Medicine and Bioinformatics, Sydney Local Health District, Royal Prince Alfred Hospital, Camperdown, NSW 2050 Australia

**Keywords:** Animal behaviour, Animal physiology

## Abstract

Sleep is important for cow health and shows promise as a tool for assessing welfare, but methods to accurately distinguish between important sleep stages are difficult and impractical to use with cattle in typical farm environments. The objective of this study was to determine if data from more easily applied non-invasive devices assessing neck muscle activity and heart rate (HR) alone could be used to differentiate between sleep stages. We developed, trained, and compared two machine learning models using neural networks and random forest algorithms to predict sleep stages from 15 variables (features) of the muscle activity and HR data collected from 12 cows in two environments. Using k-fold cross validation we compared the success of the models to the gold standard, Polysomnography (PSG). Overall, both models learned from the data and were able to accurately predict sleep stages from HR and muscle activity alone with classification accuracy in the range of similar human models. Further research is required to validate the models with a larger sample size, but the proposed methodology appears to give an accurate representation of sleep stages in cattle and could consequentially enable future sleep research into conditions affecting cow sleep and welfare.

## Introduction

Animals are driven to sleep and it is vital that enough restful sleep is achieved to feel replenished^1^. Feelings of exhaustion, tiredness and sleeplessness can impact negatively on animal welfare^2^. Health can also be significantly impacted by sleep loss (sleep deprivation or restriction), which can result in activation of the immune and inflammatory systems^3^ and also influence pain sensitivity and perception^4^ in both humans and animals.

We know very little about the importance of sleep and the effects of limited or poor-quality sleep for dairy cows. Broadly, it is likely that factors affecting lying behaviour will also influence sleep, as cows must lie down to achieve it^5^. Sleep can be affected by stressful experiences during the day^6^. Therefore, changes to sleep patterns or total sleep time in cattle could be useful indicators for stress and other welfare concerns. The ability to identify sleep stages accurately could enable research on the effects of sleep loss for cows and could be useful to inform management practices such as determining rest intervals during long-haul transport or management of cattle during wet weather (i.e. on standoff pads).

Sleep consists of two main types: rapid eye movement (REM) and non-REM (NREM) sleep. The most accurate method of identifying sleep types is polysomnography (PSG)^7,8^, which consists of a combination of physiological measurements; mainly electroencephalography (EEG), electromyography (EMG), and electro-oculography (EOG), which record electrical signals of the brain, as well as muscle and eye activity. Using specialized software, traces from these signals are analyzed and scored visually using characteristic patterns to determine sleep stages according to defined criteria. REM sleep is a deep sleep stage, where the brain is active, the muscle tone is low and there are often frequent eye movements. The majority of human total sleep time is spent in NREM sleep, which can be further divided by ‘depth’ into 3 stages from light—N1 and N2 sleep to deep N3 or slow wave sleep (SWS). SWS is characterized by high amplitude oscillating activity on the EEG accompanied by lower muscle tone and lack of eye movements. Many of the restorative functions of sleep are thought to occur in this stage^9^. Dairy cows have been found to sleep for approximately 3–4 h per day, but only around 30 min of this in REM sleep^10,11^. Therefore, most of the sleep time also consists of NREM sleep stages and it is likely that these stages serve important functions for cows as they do humans.

PSG has recently been used to record sleep in calves^12^ and cows^10^ in indoor-housed environments. However, it requires a considerable amount of training to habituate the animal to wearing the equipment, and this with intensive handling, delicate and expensive devices, specialized scoring and frequent monitoring, makes PSG impractical for large research projects on cows in uncontrolled environments such as in typical group-housed farms and outdoors on pasture. No recent studies have attempted to record non-invasive PSG of sleep of cows on pasture, probably because of the difficulty in using these instruments with cows let alone in challenging and variable outdoor conditions. An ideal solution would be an alternative method or proxy for PSG, more easily applied in a variety of environments and less intensive than PSG. As cows must lie down to sleep^5^, lying posture has been suggested as such a proxy. In calves that spend a lot more time in deep sleep stages, lying with head up and immobile and lying with the head resting on the ground or turned and resting on the flank were found to be able to estimate SWS and REM sleep time respectively^12^. However, these same postures greatly over-estimated total sleep time in adult cows^13^ and were unable to accurately detect NREM sleep. Further methods based on accelerometers to collect movement and position data from devices on the head or neck of calves and cows^14–16^ have been developed to predict sleep. However, while these models have shown some success in detecting the tucked lying posture during which most REM sleep occurs, they overestimate total sleep time and lack the ability to distinguish differences between light and deep NREM sleep, as well as wakeful inactivity. Additionally, these methods have only been validated with postural estimates of sleep and not with PSG.

During mammalian sleep, autonomic nervous activity such as heart rate^17–19^, respiration rate^20^ and body temperature change with sleep stage. Machine learning has been used to develop wearable technology for humans such as smart watches that use heart rate and activity to predict human sleep stages and duration^21^. Therefore, the potential exists to use similar physiological changes to identify different sleep stages in cows. In dairy cows, respiration rate and body temperature can be recorded for long periods of time, but are difficult^22^ or require invasive internal devices. Heart rate (HR) and heart rate variability (HRV) recording devices are relatively inexpensive and unobtrusive to the cow and can be worn for long periods of time^23,24^. Methods using machine learning to predict sleep stage from HR and HRV have been developed recently for humans^19,25^, and methods combining HR with other measures such as actigraphy further increase performance for sleep stage identification^26^.

We collected HR, lying behaviour and PSG data simultaneously from two groups of cows, housed indoors and on pasture. The aim of this project was to determine if we could accurately differentiate between different stages of light and deep sleep in dairy cows using only HR and neck muscle EMG data, compared to visual scoring of the PSG, and to compare the success of two machine learning algorithms in this task.

## Results and discussion

EEG is the recognized ‘gold standard’ to determine sleep stages however, a complicated and painstaking setup is required which makes it prohibitive to use for determining sleep stages in cows. The objective of this study was to determine the efficacy of using heart rate and neck muscle activity to determine cow sleep stages using machine learning. To our knowledge, this is the first study of its kind aimed to detect cow sleep stages using only heart and neck muscle data. Using this data alone, the machine learning models developed were able to predict 82.3% of sleep stages correctly. Classification performance of the machine learning models presented in this paper is similar to Mitsukura et al.^27^, which proposed models to detect human sleep stages using only heart rate data. Table 1 shows the values used to compare both machine learning models. The neural network (NN) analysis produced the best overall performance and had an area under the curve (AUC) value of 92.5%. Classification accuracy was 82.3%. precision was 81.5%, recall was 82.3% and F1 score was 0.814. The prediction accuracy of the NN model is just marginally better than that of random forest (RF) which produced 82.1% classification accuracy and a slightly better AUC value of 92.6%. Both neural network and random forest algorithms show the ability to learn reasonably well from the data and discriminate well between various sleep stages.

**Table 1:** Overall performance of the neural network and random forest models across all sleeping stages (average over classes) in terms of area under the curve (AUC), classification accuracy (CA), F1 score, precision, and recall (sensitivity).

Model	AUC (%)	CA (%)	F1	Precision (%)	Recall (%)
Neural network	92.5	82.3	0.814	81.5	82.3
Random forest	92.6	82.1	0.805	81.3	82.1

**Figure 1:**
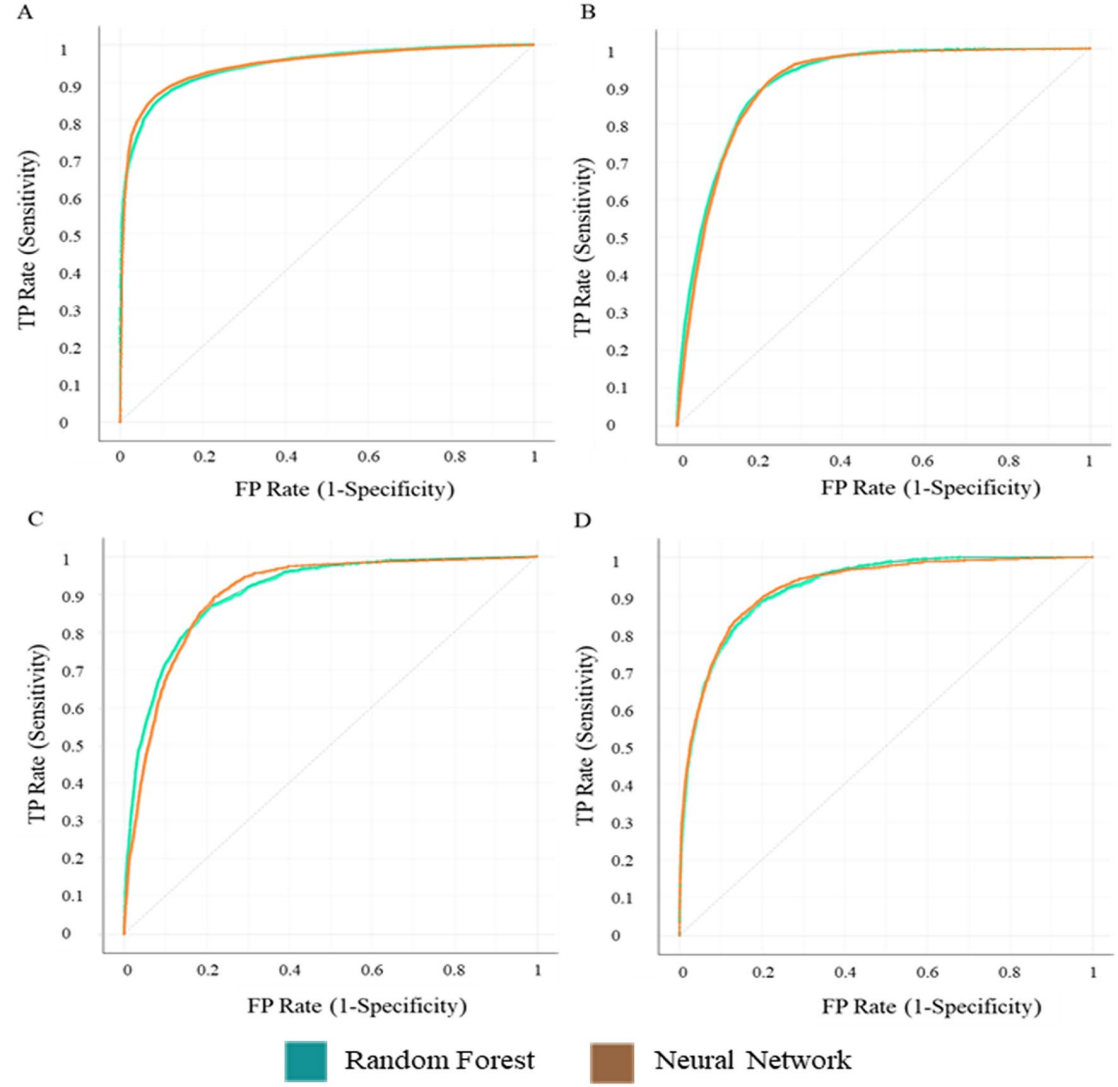
ROC curves of the Neural Network and Random forest models for detection of each individual sleep stage. (**a**) Awake stage, (**b**) combined light sleep stages N1/2, (**c**) slow wave sleep- N3 stage and (**d**) REM sleep stage. Figure created using Orange (version 3.26) https://orangedatamining.com/.

Table 2 shows the CA and AUC of both models to predict the sleep/wakes stages individually. In terms of AUC, Awake and REM stages were the most accurately detected with a 94% and 92% chance of scoring correctly. The models had slightly more difficulty identifying NREM sleep stages; however, AUC was remained at 90%. Figure 1 shows the ROC curves for the classification of each individual sleep stage by both NN and RF models. Classification accuracy for N3 and REM stages were above 95%, with awake and N1/2 ranging from 85 to 88%. Individually, N3 and light N1/2 sleep were slightly more difficult to predict according to the classification performance of various models in our dataset. As previously discussed, this could be due to errors in sleep scoring from the PSG, however NREM sleep stages are the least different from one another physiologically, so it is possible that there is a significant overlap with other sleep stages in the heart rate and neck muscle activity.

**Table 2:** Performance of both models (neural network and random forest) for individual sleep stages (awake, combined light NREM sleep (N1/2), N3 (SWS) and rapid eye movement sleep (REM)) in terms of area under the receiver operator curve (AUC) and classification accuracy (CA).

Model	Awake		N1/2		N3		REM	
	AUC (%)	CA (%)	AUC (%)	CA (%)	AUC (%)	CA (%)	AUC (%)	CA (%)
Neural network	94.7	88.4	90.8	85.2	90.2	95.3	92.4	95.8
Random forest	94.4	87.2	91.1	85.5	90.4	95.7	92.3	95.9

Our methodology involved spending a significant amount of time prior to the beginning of data collection gentling and handling the cows who had previously been unused to such an amount of human contact and training them to wear unfamiliar materials and instruments. Even with these efforts, a large amount of recorded data was then unusable due to cows rubbing electrodes off on gates, water buckets or when lying or moving, unpredictable cow behaviour, or issues with electrode impedance and the devices that could only be determined after the recording. We collected a total of 23,123 useable 30 s epochs (approximately 192 h) of PSG, HR, and activity data from a total of 12 cows in two different environments—housed indoors in the UK and on pasture in New Zealand. As there are no widely used scoring criteria for cows as there are for humans, previous work on cow sleep^10–12,28^ as well as human American Association of Sleep Medicine (AASM)2018 guidelines^29^ were used to define sleep stages. Previous cow PSG studies have only identified REM sleep, SWS and ‘drowsing’, however definitions of drowsing and implications for sleep and cow welfare are unclear^10,30^. Labelling of the sleep stages based on visual analysis of the PSG traces is accepted as common practice in human sleep scoring, however, it can be somewhat subjective and there can be a degree of disagreement even between highly experienced human sleep scoring technicians using clearly defined criteria^31^. A study of inter-rater reliability of human sleep using AASM guidelines found an overall agreement of 82.0% and Cohen’s kappa = 0.76^32^ and a study of intra-expert scoring of spindles from light sleep found agreement of 72% with k = 0.66^33^. These kappa figures suggest high, but not perfect agreement between observers. Overall intra-observer agreement for scoring sleep/awake stages from the PSG traces in this study was 89.42%, however, N1 and N2 were the least reliable as only 32% of epochs were agreed, and 39% of N1 were re-scored as N2. Combining N1 and N2 improved agreement to 91.1%. Despite an ‘almost perfect’ level of intra-observer reliability^34^, even when combining N1 and N2 stages, 8.9% of epochs were disagreed upon when re-scoring PSG. There is therefore a margin of error introduced into the model due to mistakes in scoring and labelling data from the PSG which was used as the ‘ground truth’ with which to train the model. However, with visual analysis there is always likely to be a degree of human error associated with the scoring.

Machine learning has also been used to classify sleep stages in animals such as mice^35^ and rats^36^ using spectral aspects of the EEG signals, so this could be attempted in future sleep stage labelling of cow PSG data.

Cows are ruminants and must regurgitate and re-chew their food to obtain energy. Because of their strong jaw muscle movements, distinct rhythmic chewing artefacts obscure the PSG traces making accurate identification of any potential sleep stages during rumination or chewing impossible. For this reason, epochs containing rumination were excluded from the dataset and therefore the current model is only able to identify vigilance state from data when rumination is absent. Future models could be modified to predict rumination, however sleep stage estimation during this time would be impacted by the artefacts on the EMG traces.

The data set was heavily weighted to the awake stage. As shown in Table 3, around 70% of the data set consisted of awake data with the other 30% consisting of sleep, and less than 5% of data points being in REM or N3 sleep stages. We made recordings during both day and night and included all recorded data of sufficient quality in the data set. Most cow sleep occurs at night-time, with small bouts of sleep during the day, and in total only about 4 h per day is spent sleeping^11^. Being so heavily weighted to the awake stage, the models had many more examples to learn from to identify Awake epochs, but far fewer examples from which to learn to identify N3 or REM sleep epochs. Balancing the dataset in terms of sleep and awake stages equally might help future models to learn better by having more examples of less common sleep stages.

**Table 3:** Number of data points and overall percent of data points at each sleep stage in the dataset.

Awake	16,584	71.72%
N1/2	4401	19.03%
N3	1034	4.47%
REM	1104	4.77%
Total	23,123	100%

We used 15 different features of the heart rate and EMG data and the machine learning models were able to learn from this and discriminate between various sleep stages. Classification models learn and perform well when there is a significant difference between features in various classes. Table 4 shows the rank of each feature calculated in terms of information gain (the expected amount of information or entropy), gain ratio (a ratio of the information gain and the attribute’s intrinsic information, which reduces the bias towards multivalued features that occurs in information gain) and ANOVA (the difference between average values of the feature in different classes). The features of our dataset that were the most informative for the machine learning models were mainly the Neck EMG features (Neck RMS, Neck Variance, and Neck Standard Deviation). The highest scoring features of our dataset were the Neck EMG features (Neck RMS, Neck Variance, and Neck Standard Deviation). A reduction of muscle tone in the neck muscles is a classical indicator used for the visual identification of REM sleep from PSG data. The higher AUC and accuracy values for the prediction of REM sleep compared to other sleep stages may be due to the high rank of the neck EMG features (Table 2). Mitsukura et al.^27^ predominantly used frequency domain features of the HRV signal for sleep stage classification in humans, and it is possible that frequency domain features could be useful for cow sleep staging as well. However, we only used time domain features of the HRV as we were working from 30 s epochs, which is arguably too short of a window to calculate frequency domain metrics from. Frequency metrics are usually calculated for 5 min periods, and while it could be possible to increase epoch size to 5 min to allow for these calculations, this would reduce the granularity and possibly result in longer epochs containing several sleep stages as some bouts of individual stages have durations of less than 2 min. Long epochs consisting of multiple stages could also introduce confusion into the model resulting in more misclassification.

**Table 4:**
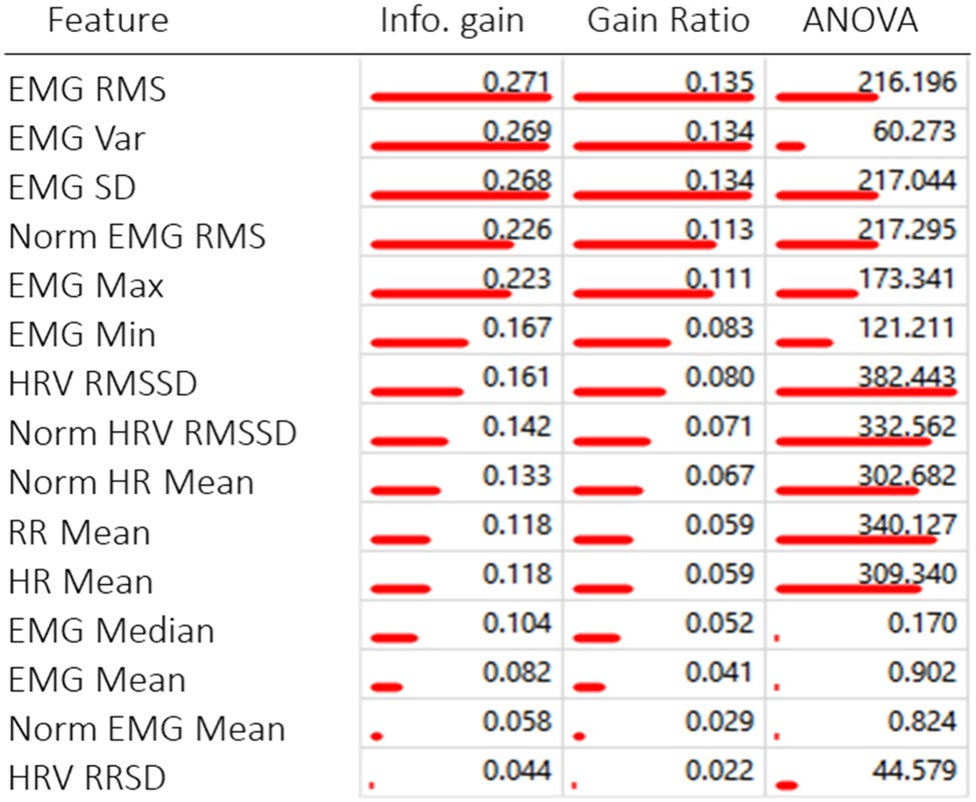
Ranking of features in the dataset from overall most informative to least and ranking by each calculation; info gain, gain ration and ANOVA (redlines).

Table produced using Orange (version 3.26) https://orangedatamining.com/.

The classification models were developed with data from two separate groups of cows which were different in terms of breed, age, housing, and previous experience. All cows were non-lactating, but the Kiwi-cross (NZ) cows were also in mid-late pregnancy during the recording period. There were differences between the two populations in terms of average HR and the Kiwi-cross cows generally had a higher heart rate than the UK group. These differences could be due to age, size of cows and pregnancy status, but highlights the possibility of hidden batch effects within the model. More training data from different populations of cows, and cows in different stages of lactation would be beneficial to increase confidence in the classification ability of the model.

Sleep in mammals typically occurs in cycles with REM sleep following a bout of NREM however, NREM sleep can also occur on its own^37^. Sleep is regulated homeostatically, but achieving a certain amount of REM sleep does not necessarily mean that a proportionate amount of NREM will also be achieved^37^. In the development of the models, we considered each 30 s epoch independently, however, they are in a time series and make up bouts lasting from a few minutes to a few hours. Preceding epoch classification therefore could have an influence on the classification decision for the successive epoch. Information on typical cow sleep patterns and bout lengths could possibly aid in future models to predict sleep stages in cows.

The current model is a marked improvement over sleep staging models for cows using only accelerometers to predict NREM and REM developed in the past that were only able to predict up to 70% of sleep correctly^14^. These models also used behavioural observations to label sleep stages, which has been shown to overestimate sleep in cows^13^. Our models have been developed with sleep stages labelled using PSG rather than behavioural observations, and while not as simple as accelerometers, EMG and HR monitoring equipment are small and far easier to use with cows than a full PSG montage.

We investigated the use of non-invasively acquired EMG and HR data to predict sleep stages from light N1/2 sleep to deep N3 and REM sleep in dairy cows. While these models have been developed with a small sample size, our classification models developed with Neural Network and Random Forrest algorithms achieved similar outcomes, both with good accuracy, suggesting neck EMG and HR data could be suitable to predict sleep stage with some reliability in dairy cows. More data from cows of different breeds, ages and lactation stages would be beneficial to improve future models. We believe the use of HR and Neck EMG is promising for future identification of sleep stages in dairy cows from non-invasive physiological recording devices. This will enable future research into the effects of typical husbandry practices, transport and environment on cow sleep and the importance of sleep for cow health and welfare.

## Methods

### Animals and on farm management

Ethical approval for all procedures involving animals was obtained from the UK Home Office (Project Licence P204B097E), SRUC Animal Ethics Committee (Ref. ED AE 03-2018) and Ruakura Animal Ethics committee (AE 14708) prior to study onset. All methods were carried out in accordance with UK and New Zealand animal welfare guidelines and regulations and the authors have complied with the ARRIVE guidelines.

**Figure 2:**
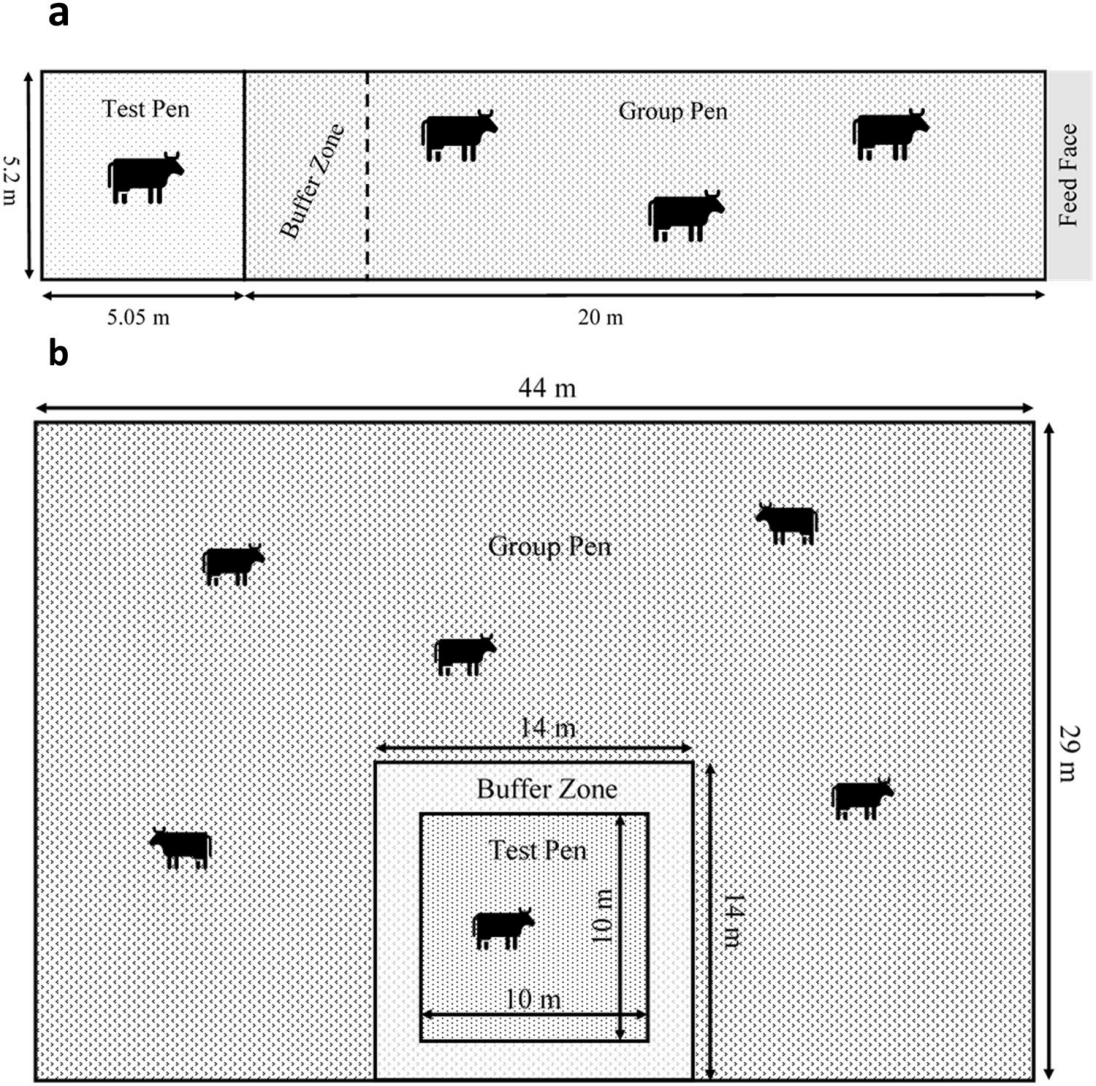
Diagrams of group and test pen design in the UK indoor housed study (**a**) and in the NZ outdoor pasture study (NZ) (**b**). During recordings, the test cow was moved into the test pen, when not recording, the cow was moved back into to the group pen.

The indoor study was conducted with 6 non-pregnant, Holstein cows (average age 3.86 ± 0.68 years) who were selected from the herd at SRUC Acrehead Farm (Dumfries, Scotland) based on farm staff knowledge of their approachable nature. When enrolled, the cows were either non-lactating or dried off according to routine farm practice prior to the study and were housed in a 20 m × 5 m group pen, deep bedded with straw, within the main barn and fed as per routine farm practice. A 5 m × 5 m test pen was located adjacent to the group pen, but could be separated by a buffer zone of approximately 2 m to reduce potential damage to recording equipment and disruptions to the recordings of other cows, while maintaining visual and auditory contact with the group (Fig. 2).

The protocol was then repeated at pasture with six approachable, mid-late pregnant, non-lactating three-year-old Kiwi-cross (Friesian-Jersey) cows selected from the herd at DairyNZ Lye Farm (Newstead, NZ). These cows were managed outdoors in a large (44 m × 29 m) group pen created with electric fencing that could be moved around within a larger paddock as ground conditions deteriorated. A 10 m × 10 m test pen was created with non-live electric fencing (to reduce potential electrical noise on physiological traces) on one side of the group pen. A 2 m buffer zone with live electric fencing was set up around the test pen, allowing for visual and auditory contact of the test cow with the group at all times (Fig. 2). Cows were allowed to graze and were supplemented with silage ad libitum. All cows in both groups were trained and habituated to the recording devices and handling protocols for a minimum of 2 weeks prior to the start of data collection.

### Data collection methods

#### Polysomnography

**Figure 3:**
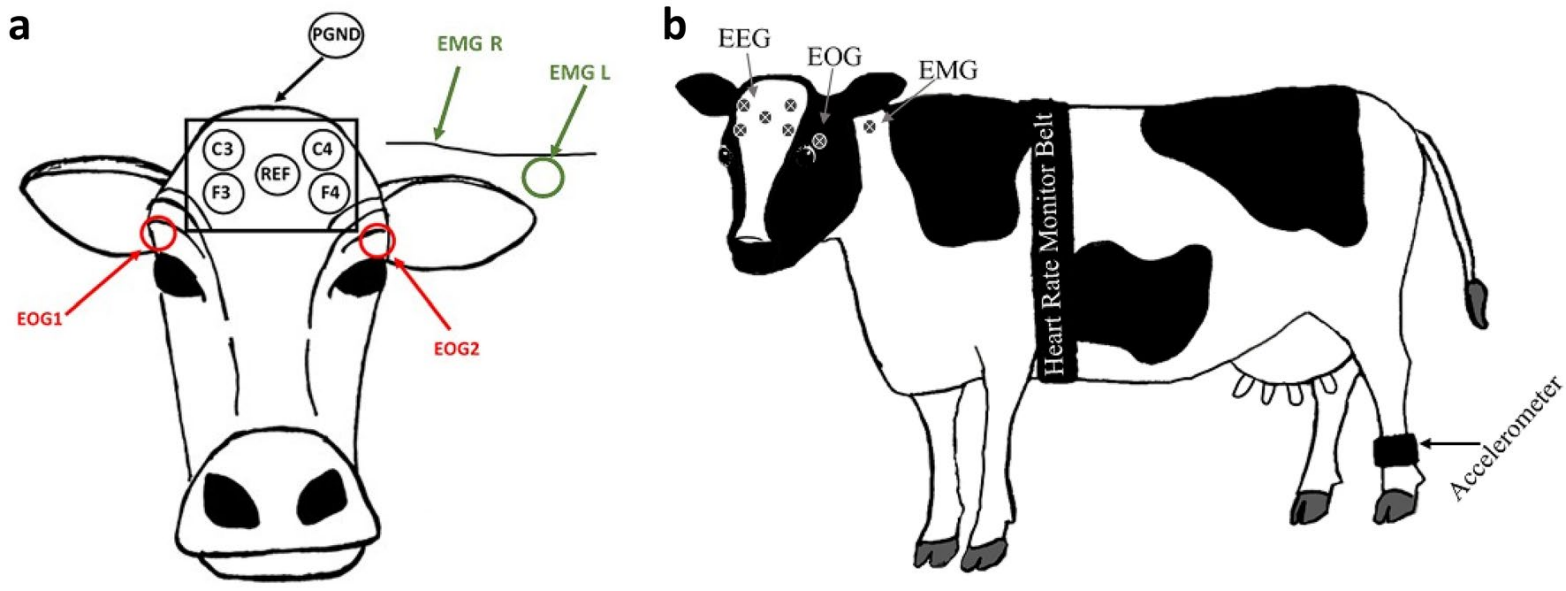
(**a**) Diagram indicating electrode placement on the head and neck of the cow for PSG data acquisition. Four EEG electrodes (C3, C4, F3 & F4) and a reference (REF) electrode were placed on the forehead. PGND- patient grounding electrode was placed behind the poll on the top of the head. Two EOG electrodes were placed beside the eyes and two EMG electrodes were placed on the mid-trapezius muscle on either side of the neck. (**b**) Diagram indicating placement of the heart rate monitoring girth strap, leg mounted accelerometer, and PSG electrodes on the whole cow.

PSG was recorded using a 10 electrode montage as described in Hänninen et al.^12^. This included 4 EEG, 2 EOG and 2 EMG electrodes as well as a ground and reference electrode attached to the head and neck of the cow (Fig. 3). Adhesive pre-gelled ECG electrodes (Natus neurology, Kanata, Canada) were used and secured to clipped and cleaned skin on the head and neck of the cow with a small amount of superglue (Loctite 454 or Loctite gel control, Henkel Corp., Dublin, Ireland). A stretchable LeMieux^®^ or Caribu Lycra horse hood (UK: Horse Health Wessex, Woodington, UK. NZ: Caribu AU, Truganina, Australia) was modified for the cow anatomy and worn on the head and neck over top of the electrodes to keep all wires close to the skin and avoid being tangled in the test pen. After data collection was completed, all materials were removed and electrodes either came away easily or were gently removed using acetone or aqueous cream to soften the glue. Signals were sampled at 500 Hz and recordings ran for 10 h due to memory capacity of the Embletta MPR PG + ST proxy recording device (Embla, Natus Neurology, Kanata, Canada). The recording device was programmed, and data were downloaded using RemLogic 3.4.3 software (Embla Systems, Kanata, Canada). After downloading, a 50 Hz mains filter was applied to all traces to remove the background noise caused from electrical wires that are present in the environment and can be picked up by the PSG device, in the UK and NZ electrical mains frequencies are both at 50 Hz. EEG traces were high pass and low pass filtered at 0.3 Hz and 30 Hz, EOG traces were filtered at 0.15 Hz and 20 Hz and EMG at 10 Hz. Traces were first inspected for quality, “good” quality recordings included those where impedance was within the acceptable range (> 14 Ω) and at least 2 EEG, 1 EMG and 1 EOG trace remained attached for the entire recording period. “Poor” quality recordings were not scored and occurred when impedance was too high, there was noise on the traces, many artefacts obscured the data or electrodes became detached during the recording. Traces were then scored visually in 30 s epochs into 4 stages of sleep (N1, N2, N3, REM), wakefulness (W) and rumination (RNT) by a single scorer trained in human sleep staging, according to staging criteria developed from previous work on cow sleep^10–12,28^ as well as human American Association of Sleep Medicine 2018 guidelines^29^.

#### Heart rate

Heart rate (HR) and inter-beat intervals (between R peaks of the heart beat signal: (R-R)) were recorded using a Polar equine monitoring girth strap with electrodes near the heart and the reference electrode near the shoulder (Fig. 3) and logged with the Polar RS800CX Watch (Polar Electro Oy, Kempele, Finland). The time was synchronized between the watch and PSG recording devices. Ultrasound gel (Aquasonic 100 gel, Parker Laboratories, NJ, USA) was applied liberally at electrode locations. Data were downloaded using Polar Pro-Trainer 5 software (Polar Electro Oy, Finland). After downloading, the signal was filtered using Polar Pro-trainer 5 at a moderate filter power with a minimum protection zone of 6 bpm. Only traces containing less than 1% identified errors were used for analysis. The filtered data were then extracted, and statistics were calculated in 30 s epochs corresponding to the timestamps of the PSG epochs. Only the time domain metrics of the heart rate variability were calculated, as the validity of frequency domain metrics in intervals smaller than the 5 min standard are questionable^38^.

#### Lying behaviour

Lying and standing times were recorded continuously using an accelerometer (UK; IceTags (Ice Robotics, Edinburgh, Scotland), NZ; Onset Pendant G data loggers (64 k, Onset Computer Corporation, Bourne, MA) attached on the lower hind leg (Fig. 3). The data were downloaded using IceManager Software (Ice Robotics, Edinburgh, Scotland) or HOBOware Pro software (Onset Corp., Pocasset, MA). Lying and standing behaviour were determined from the data-logger files in 30 s epochs corresponding to the PSG epochs.

### Data pre-processing and segmentation

Neck muscle activity data was extracted from a single good quality EMG trace per recording. Statistics were calculated for each epoch, including mean, maximum (max), minimum (min), median (med), standard deviation (SD), variance (Var) and root mean square (RMS) using RemLogic software.

Mean HR, mean R-R interval, Standard deviation of RR intervals (SDRR) and Root Mean Square of Successive Differences (RMSSD) (Eq. 1) were calculated from the exported and filtered polar heart rate data for each 30 s epoch corresponding to the PSG epochs.

1 $$RMSSD= \sqrt{\frac{1}{N-1}\left({\sum_{i-1}^{N-1}{(\left(RR\right)}_{i-1}-{\left(RR\right)}_{i})}^{2}\right)}$$

Normalized HR mean, RMSSD, EMG mean, and EMG RMS values were also calculated by dividing the data by the largest point for each individual recording as a way of removing some of the variation between cows and between recordings. All 15 parameters or ‘features’ from the HR, HRV, EMG and lying behaviour data were merged and matched with the scored sleep stage epochs using R Studio (Version 1.3.959) using time stamps and epoch numbers.

**Figure 4:**
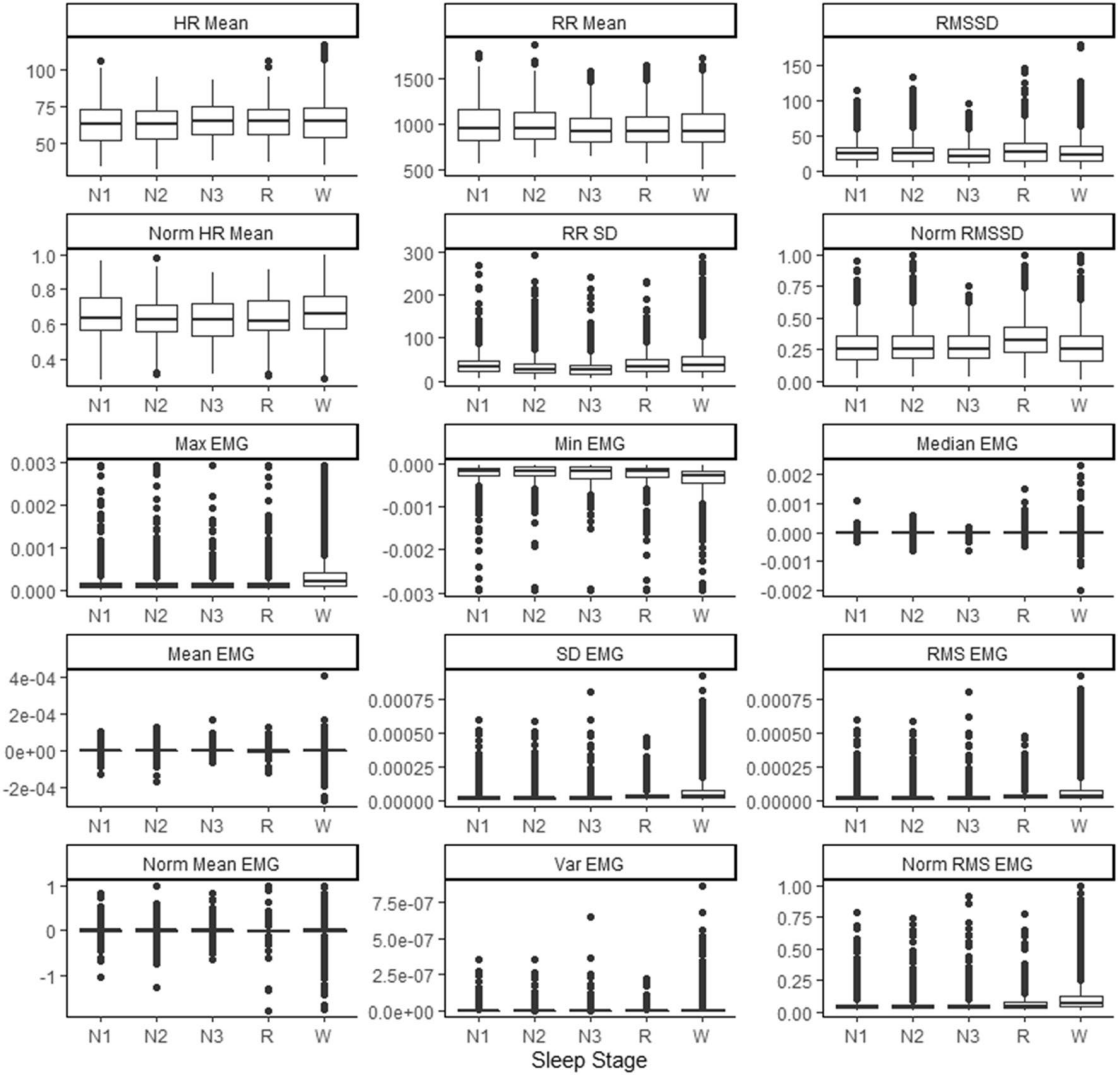
Box and whisker plots of each feature (titles), with sleep stage on the x-axis and relevant units on the y-axis. The y-axis of HR mean, and Norm HR mean are expressed as beats per minute (BPM), while RMSSD, RRSD and the normalized graph of these are expressed in milliseconds. The y-axis of all EMG graphs is expressed in microvolts (µV)Figure produced in R version 4.0.2 using ggplot2 package https://cran.r-project.org/web/packages/ggplot2/index.html.

Intra-observer reliability was calculated using Cohen’s kappa in the “irr” package in R (Version 4.0.2). Overall agreement was 89.4% with k = 0.83however, N1 and N2 were the least reliable as only 32% of epochs were agreed, and 39% were misidentified as N2. In exploration of the physiological data, N1 and N2 were not vastly visually different in terms of mean and variance (Fig. 4), and so were combined into a new stage of light sleep named ‘N1/2’- to improve classification performance. Combination of N1 and N2 improved overall agreement to 91.1% (k = 0.86). Rumination causes rhythmic chewing activity artefacts that obscure the PSG traces and make it impossible to determine brain activity and sleep stage. It is possible that cows could achieve sleep during rumination, which could create confusion and misclassification of the data, so for this reason it was removed from the data set. Dairy cows must lie down to sleep^5^, therefore epochs determined as ‘standing’ from the accelerometer data were also removed from the data set.

From visual and exploratory analysis of the data set, there were no clear differences between sleep stages for any of the features. There were minor differences such as REM sleep tending to have a higher RMSSD than other sleep stages, and the means of W were higher for max EMG, RMS EMG and SD EMG than for the other sleep stages (Fig. 4).

Altogether there were 23,120 data points labelled into 4 different sleep stages (Awake, N1/2, N3 and REM) each with corresponding data from the 15 different features (HR Mean, RR Mean etc.). Table 3 shows number of data points for each sleep stage, the awake category has the greatest number (16,584) of data points, while the combination of N1 and N2 (N1/2) had 4401 data points, REM had 1104 and N3 had 1034 data points.

### Machine learning method for sleep stages

To predict cow sleep stages using only heart and neck muscle data, we considered two machine learning techniques: Neural Network^39^, and Random Forest^40^. Both the machine learning models were implemented using the open source Orange machine learning platform (Version 3.26)^41^. Stratified tenfold cross-validation was used to train and test the models.

Architecture of the Neural Network Model:

- Number of neurons in hidden layers: 500.
- Activation function: ReLu.
- Solver: Adam.
- Regularization: 0.0001.
- Maximal number of epochs/iterations: 2000.

During the cross-validation process, the whole dataset was randomly split into a labelled or ‘known sleep stage’ data set to train the model with, the remaining data having the labels hidden and used to test the model with. For example, REM had 1104 observations, approximately 110 observations were used for testing and rest were used for training and this process was repeated 10 times for each sleep stage. The model’s predictions were then compared with the actual labelled sleep stages to test and compare the models. Classification accuracy (CA) (the number of correct predictions divided by the total number of predictions), recall (sensitivity or true positive rate), precision (a measure of the model’s exactness), F1 score (the balance between Precision and Recall) and area under the curve (AUC) determined from the receiver operator curve (ROC) values from each model were used to measure the performance. The classification accuracy (Eq. 2), precision (Eq. 3), recall (Eq. 4) and F1 score (Eq. 5) were obtained from true negative (TN), false negative (FN), true positive (TP), and false positive (FP) values. This process was repeated for 10 random splits or ‘folds’ and classification accuracy of each machine learning technique was measured by taking the average across the 10 folds.

2 $$Classification \; Accuracy\,(CA)=\frac{TP+TN}{TP+TN+FP+FN}$$

3 $$Precision=\frac{TP}{TP+FP}$$

4 $$Recall=\frac{TP}{TP+FN}$$

5 $$F1 \;Score=\frac{2TP}{2TP+FP+FN}$$
